# Content validity and psychometric evaluation of the Functional Assessment of Chronic Illness Therapy-Fatigue scale in patients with chronic lymphocytic leukemia

**DOI:** 10.1186/s41687-021-00294-1

**Published:** 2021-03-11

**Authors:** Daniel Eek, Cristina Ivanescu, Laura Corredoira, Oren Meyers, David Cella

**Affiliations:** 1 AstraZeneca Gothenburg, Pepparedsleden 1, 431 50 Mölndal, Sweden; 2 IQVIA, Amsterdam, The Netherlands; 3 IQVIA, Barcelona, Spain; 4 IQVIA, New York, NY USA; 5 Northwestern University, Evanston, IL USA

**Keywords:** Chronic lymphocytic leukemia, Content validity, FACIT-Fatigue, Psychometric properties

## Abstract

**Purpose:**

Fatigue is a prominent symptom in individuals with chronic lymphocytic leukemia (CLL). This work evaluates the content validity and psychometric properties of the Functional Assessment of Chronic Illness Therapy-Fatigue scale (FACIT-Fatigue) in patients with CLL to determine if it is fit for purpose in CLL research.

**Methods:**

The FACIT-Fatigue yields a 13-item total score from a five-item symptom subscale and an eight-item impact subscale. To evaluate content validity, cognitive debriefing interviews were conducted with 40 patients with CLL in the first-line or relapsed or refractory setting. Psychometric properties, including structural validity, internal consistency, construct and known-groups validity, were investigated using data from a phase 3 trial in relapsed or refractory CLL (NCT02970318).

**Results:**

Interviewed patients considered the FACIT-Fatigue items relevant to their CLL experience, understood the terminology and agreed with response options. Confirmatory factor analysis confirmed the presence of symptom and impact subscales, but also supported unidimensionality of the FACIT-Fatigue. The FACIT-Fatigue total, symptom and impact subscales demonstrated good internal consistency (Cronbach’s coefficient α > 0.85 and McDonald’s omega ω > 0.90), and strong correlations with relevant EORTC QLQ-C30 scales (all Spearman’s *r* ≥ 0.5). Known-groups validity was shown by significant differences between groups defined by baseline performance status, hemoglobin level and constitutional symptoms (all *p* < .0001). Cluster analysis supported FACIT-Fatigue score thresholds of 30 and 34 to define a severe fatigue population.

**Conclusions:**

Content validity and psychometric evaluation in patients with CLL demonstrated that the FACIT-Fatigue has good psychometric properties and is fit for purpose in CLL.

**Supplementary Information:**

The online version contains supplementary material available at 10.1186/s41687-021-00294-1.

## Introduction

Chronic lymphocytic leukemia (CLL) is a long-term disease that typically develops and progresses slowly. In patients with CLL, abnormal lymphocytes accumulate in the blood, bone marrow and lymphatic tissues over time, resulting in anemia, bleeding and increased susceptibility to infections [1, 2]. Fatigue is one of the main symptoms of hematological cancers such as CLL, and is thought to be caused by underlying anemia and pathophysiological pro-inflammatory disease mechanisms [3]. In patients with cancer, moderate to severe anemia (hemoglobin [Hb] levels < 110 g/L [4]) has been shown to be associated with persistent fatigue and generalized weakness [3], and anemia severity correlates with the degree of fatigue [5].

In a recent qualitative interview study, patients with CLL reported fatigue as a key symptom and impact of their disease and its treatment [6]. The manifestations of fatigue included a lack of energy, weakness, decreased physical functioning and a reduced ability to maintain professional and social roles [6]. The importance of fatigue as a central symptom and impact of CLL, and as a potential indicator of disease severity, makes it relevant to measure in clinical trials assessing new treatments for CLL. The Functional Assessment of Chronic Illness Therapy-Fatigue scale (FACIT-Fatigue) is a patient-reported outcome (PRO) instrument designed to assess fatigue-related symptoms and their impact on daily functioning. The FACIT-Fatigue comprises a five-item symptom subscale and an eight-item impact subscale; in total, the scale includes 13-items. Previous work in general and cancer population samples indicates that the fatigue experience and the impact of fatigue are aligned on one single dimension and supports the FACIT-Fatigue total score as a reasonable endpoint choice for clinical research [7]. However, factor analysis has also indicated that the two subscales can be employed and reported separately, should this be needed in specific settings [7]. To be fit for purpose, PRO instruments need to have documented reliability and validity in the intended target patient population [5]. The FACIT-Fatigue has extensive published evidence of its reliability and validity in patients with cancer [7–11], although not specifically with CLL.

The current study evaluates the content validity and psychometric properties of the FACIT-Fatigue in patients with CLL to determine if it is fit for purpose in this population.

## Methods

Content validity of the FACIT-Fatigue was evaluated in qualitative interviews with CLL in the first-line (1 L) setting or in the relapsed or refractory (R/R) setting. The interviews included cognitive debriefing of the instrument. Reliability and validity of the FACIT-Fatigue were assessed in patients with R/R CLL enrolled in a phase 3 trial assessing acalabrutinib in R/R CLL (ASCEND; NCT02970318) [12].

### Measures

#### FACIT-fatigue

The FACIT-Fatigue includes a five-item symptom subscale and an eight-item impact subscale that together make up the 13-item total score. Item responses range from 0 (‘not at all’) to 4 (‘very much’). Scores for negatively worded items are reversed, such that higher scores are better (i.e. less fatigue). The FACIT-Fatigue total score ranges from 0 to 52 (the general population mean score is 43 [5, 13]). The recall period for each item is the past 7 days.

#### EORTC QLQ-C30

The European Organisation for Research and Treatment of Cancer Quality of Life Questionnaire-Core 30-questions (EORTC QLQ-C30) contains five multi-item function scales (physical, role, cognitive, emotional, social), three symptom scales (fatigue, pain, nausea/vomiting), five single-item symptoms (dyspnea, insomnia, appetite loss, constipation, diarrhea), a global health status scale and a single-item financial impact question. High function scale or global health status scale scores represent a high level of functioning and a high quality of life, respectively, whereas a high symptom score 'represent' a high level of symptomatology/problems.

#### EQ-5D-5L and EQ-VAS

The 5-level, 5-dimension EuroQol questionnaire (EQ-5D-5L) comprises five impairment-related dimensions (mobility, self-care, usual activities, pain/discomfort, anxiety/depression). Each dimension is defined from 1, indicating no problem, to 5, indicating extreme problems. Its global health visual analogue scale (EQ-VAS) is a 0–100 scale of a patient’s health status, where 0 represents the ‘worst health you can imagine’ and 100 the ‘best health you can imagine’.

### Cognitive debriefing interviews

As part of a qualitative interview study [6], cognitive debriefing interviews were conducted with 40 patients with 1 L CLL or R/R CLL resident in the United States. Full methods and results of the concept elicitation part of the interview study have been published previously [6]. Potential participants were identified via a patient advocacy organization (CLL Society; https://cllsociety.org) and two market research firms (Liberating Research, www.liberatingresearch.com; and Rare Patient Voice, https://rarepatientvoice.com), and were contacted by email and telephone about study details and participation. To be eligible, patients needed to be aged 18 years or older, be diagnosed with CLL, have a self-reported Eastern Cooperative Oncology Group (ECOG) Performance Status score ≤ 2, be proficient in English and have experienced at least one constitutional symptom of CLL (fatigue, weight loss, fever or night sweats) in the past week. Patients in the R/R CLL group had to have received two or more lines of treatment specifically to treat CLL.

The qualitative interviews were carried out by telephone and generally lasted 60–75 min in total for the concept elicitation and cognitive debriefing parts combined. Interviews were conducted by trained interviewers (O. Meyers, C. Krogh, S. Lee; IQVIA). Patients completed the FACIT-Fatigue as part of cognitive debriefing. During cognitive debriefing, participants were asked to review the FACIT-Fatigue. Patients’ observations of the FACIT-Fatigue were grouped by feedback on the instrument instructions (clarity, difficulty understanding), individual items, response options and the questionnaire as a whole (missing and redundant items).

De-identified transcripts of patient interviews were coded using ATLAS.ti software (version 8). Two coders, who had also moderated most of the patient interviews, coded the results of and feedback on the FACIT-Fatigue. Inter-coder agreement was assessed periodically throughout the coding process, and any disagreement was discussed and addressed.

### Psychometric analysis in CLL

Data for the psychometric analysis of the FACIT-Fatigue were from baseline assessments in the phase 3 ASCEND trial (NCT02970318), a multicenter, open-label study that enrolled patients with R/R CLL [12]. Eligible patients were aged 18 years or older, had previously been treated with at least one systemic therapy and had an ECOG Performance Status score ≤ 2. Patients were randomized 1:1 to acalabrutinib 100 mg twice daily or investigator’s choice of therapy (either idelalisib 150 mg twice daily plus rituximab [375 mg/m^2^ intravenously on day 1 of cycle 1, then 500 mg/m^2^ intravenously every 2 weeks for 4 doses and thereafter every 4 weeks for 3 doses] or bendamustine 70 mg/m^2^ intravenously on day 1 and 2 of each 28-day cycle plus rituximab [375 mg/m^2^ intravenously on day 1 of cycle 1, then 500 mg/m^2^ intravenously on day 1 of cycles 2–6]).

Patients in the ASCEND trial completed the FACIT-Fatigue, EORTC QLQ-C30, EQ-5D-5L and EQ-VAS at baseline and during the study. Mean total, subscale and item scores were calculated. The presence of floor effects (> 25% of patients scoring ‘worst possible health state’) and ceiling effects (> 25% of patients scoring ‘best possible health state’) was assessed.

#### Confirmatory factor analysis

Confirmatory factor analysis was employed to evaluate the latent structure (i.e. underlying subscales) of the FACIT-Fatigue instrument. First, a single factor model of the FACIT-Fatigue was examined to determine the unidimensionality of all 13 items of the instrument. If the model fits the data well and all item factor loadings are greater than 0.3, the FACIT-Fatigue can be considered unidimensional [7]. Next, a bifactor model was examined [14–16]. The bifactor model comprised a general factor of all 13 items, and two sub-domain factors, which were defined by the five symptom items and eight impact items, respectively. If all general factor item loadings are greater than 0.3 and loadings are higher on the general factor than they are on the sub-domains, the general factor can then be considered measurable even in the presence of sub-domain factors [7].

Unidimensionality was evaluated by examining fit statistics of the confirmatory factor analysis (CFA) models and the investigation of factor loadings to assess the relative impact of the secondary dimensions. The following fit indices were evaluated: the root mean square error of approximation (RMSEA) [17]; standardized root mean square residual (SRMR) [18]; comparative fit index (CFI) [19]. The RMSEA and SRMR measure the discrepancy between the observed sample and the hypothesized model. The CFI is an incremental fit index with the null hypothesis that all components in the model are uncorrelated. In addition, factor loadings from single factor modeling and loadings on the general factor of the bifactorial model were compared to assess the level of disturbance due to multidimensionality in the data [20]. Standard cutoff values were used for RMSEA (< 0.06), SRMR (< 0.08) and (CFI > 0.95) [17–20].

Model identification was ensured by restricting the factor variance to 1, making sure that at least three indicator variables per latent factor were considered and verifying that the number of datapoints was larger than the number of parameters to be estimated. Mplus 8.0 was used to perform the factor analyses. We employed the mean and variance-adjusted weighted least-squares (WLSMV) estimator, suitable for the analysis of categorical data, polychoric correlations and theta parameterization – in which residual variances of observed categorical outcome variables are allowed to be parameters in the models [21]. A pairwise present approach to missing data was used as it is the default in Mplus with WLSMV estimator [22].

#### Internal consistency reliability

Internal consistency reliability is a measure that summarizes the correlations across instrument items. Cronbach’s coefficient α was used to assess internal consistency reliability of the FACIT-Fatigue symptom subscale, the impact subscale and the total scale. A Cronbach’s coefficient α ≥ 0.70 indicates acceptable reliability [23]. In addition, McDonald’s omega (ω) and omega hierarchical (ωH) coefficients were calculated as they provide better estimates of measurement precision (reliability) than the traditional Cronbach’s alpha [24]. Omega coefficients estimate the proportion of variance in unit-weighted total score attributable to all sources of common variance and to the general factor within the bifactor framework [16, 25, 26]. A high ω value suggests a highly reliable multidimensional composite and a high ωH value (> 0.80), when a bifactor structure is employed, suggests that the general factor is the dominant source of systematic variance with sub-domain factors having less influence. The unidimensionality of the index was also evaluated by calculating the Explained Common Variance index (ECV) [27, 28]. Higher values of ECV indicate a strong general factor allowing us to fit a unidimensional model even to multidimensional data.

#### Construct validity

Construct validity examines the relationship among scales that measure similar concepts (convergent validity) and among scales that measure different concepts (divergent validity). Convergent validity and divergent validity were assessed to explore associations between the FACIT-Fatigue and the EORTC QLQ-C30 and EQ-VAS, using Spearman’s rank correlation coefficients. Spearman’s rank correlation coefficients ≥ 0.50 were considered to demonstrate convergent validity; Spearman’s rank correlation coefficients < 0.30 demonstrated divergent validity [29]. Moderate to high correlations were expected between the FACIT-Fatigue scores and the fatigue scale from the EORTC QLQ-C30, supporting convergent validity. Although fatigue is likely to affect most aspects of quality of life, low correlations were expected between the FACIT-Fatigue scores and gastrointestinal-related scales (e.g. constipation) from the EORTC QLQ-C30, supporting divergent validity.

#### Known-groups validity

Known-groups validity is a form of construct validity that explores if scales differentiate between groups that are hypothesized a priori to differ. FACIT-Fatigue scores between groups known to be different were compared using analysis of variance (ANOVA) on baseline data. Known groups comparisons were explored based on ECOG Performance Status score (0 [fully active] vs 1 or 2 [restricted activity but still ambulatory and capable of all selfcare]), Hb level (≥ 110 g/L [no/mild anemia] vs < 110 g/L [moderate/severe anemia] [4]) and constitutional symptoms (night sweats, fever, unexplained weight loss, significant fatigue [none vs ≥ 1 symptom]). It was hypothesized that patients with an ECOG score ≥ 1, with moderate to severe anemia or with constitutional symptoms would have lower FACIT-Fatigue scores (more fatigue) than patients with an ECOG score of 0, no moderate to severe anemia or with no constitutional symptoms. The following baseline covariates were included: sex (male vs female) and age. Because so few patients had an ECOG Performance Status score of 2, the known groups employed here differ from the stratification of 0 or 1 vs 2 which was used as part of the stratified randomization in the ASCEND trial.

#### Defining the severity cut-off score

Severity cut-off scores were explored for differentiating between patients with low symptom levels and those with higher symptom levels, to define a severe fatigue population. Cluster analysis was performed to identify a FACIT-Fatigue severity cut-off score [30]. Clusters were formed using the FACIT-Fatigue symptom subscale and EORTC QLQ-C30 Fatigue scale scores on one hand, and using individual FACIT-Fatigue and EORTC QLQ-C30 item scores on the other hand. Scores were first standardized on their ranges to equalize the influence of variables with different scale lengths on the cluster solution. A two-step cluster analysis using SPSS [31] was then used to determine the cluster membership. An analysis by Cella et al. suggested one standard deviation (SD) below the general population mean of 43 (SD: 9) to denote the threshold for fatigue impairment, resulting in a cut-off value of 34 [5]. In addition, a FACIT-Fatigue threshold of 30 for fatigue impairment, suggested by Piper and Cella [32], was also considered in our analysis. Agreement between the clusters and thresholds was assessed using Cohen’s kappa coefficient [33]. Cohen characterized values ≤ 0 as indicating no agreement, and 0.01–0.20 as slight, 0.21–0.40 as fair, 0.41–0.60 as moderate, 0.61–0.80 as substantial and 0.81–1.00 as almost perfect agreement.

## Results

### Cognitive debriefing interviews

The interview study included 20 patients with 1 L CLL and 20 patients with R/R CLL. Median age of participants was 58 (range: 28–73) years, and sex distribution was approximately equal (men: 52%; women: 48%). The mean FACIT-Fatigue score among the patients participating in the qualitative interview study was 28.9 (SD: 13.6) for patients with 1 L CLL and 29.3 (SD: 11.5) for those with R/R CLL, out of a possible maximum score of 52. All patients confirmed that the FACIT-Fatigue was reflective of their experiences with CLL-related fatigue. Patients confirmed that most of the terminology was clear and well understood, and that the wording frequently reflected that used by patients with CLL-related fatigue. Table 1 shows a selection of patients’ feedback on the FACIT-Fatigue.

**Table 1:** Selection of feedback on the FACIT-Fatigue from patients with CLL

Characteristics of FACIT-Fatigue	Feedback from patients
Clarity of instructions and items	*“I think they’re clear and I think that they’re valid.” (Patient with 1 L CLL)* *“Straightforward, and easy, and clear.” (Patient with 1 L CLL)* *“I think these questions are all pretty well designed.” (Patient with R/R CLL)*
Suitability of response options	*“There are enough options. It is better than saying yes or no.”* *“I think [the distinctions between 0 and 4] are pretty clear.”*
Relevance to patients with CLL	Overall *“I think it’s pretty thorough. It states everything from activities to the energy to the eating, a lot of things that I know I’ve struggled with.” (Patient with 1 L CLL)* *“I think these questions are spot on [in making people understand my fatigue].” (Patient with 1 L CLL)* Item 10 (“I am too tired to eat”) *“I can’t imagine anybody being too tired to lift a fork, but I feel if you’re too tired to eat, it’s probably because you’re physically exhausted, and there is some effort involved in making or getting food for yourself that you just don’t want to put the effort in.” (Patient with R/R CLL)* *“That’s very clear and probably very pertinent for somebody who’s really that tired. I’m sure it happens.” (Patient with R/R CLL)* Item 3 (“I feel listless/washed out”) *“This one was a little unclear. Washed out is not a term that I use or I’m familiar with. When I think of listless, I think of need something to do. Almost bored or fidgety.” (Patient with 1 L CLL)*

*Abbreviations: CLL* chronic lymphocytic leukemia, *FACIT-Fatigue* Functional Assessment of Chronic Illness Therapy-Fatigue scale, *1 L* first-line setting, *R/R* relapsed or refractory setting

Patients considered the FACIT-Fatigue items to be relevant to patients with CLL and distinct from each other, although the item ‘I am too tired to eat’ was not considered to be highly pertinent. Respondents could imagine that some patients with CLL might be too tired to eat, but they recalled being too tired to *prepare* a meal, rather than too tired to eat it. However, respondents felt that the item was still relevant and that it did not detract from the applicability or clarity of the FACIT-Fatigue. Fatigue-related impact items were thought to capture adequately both the mental and physical impacts of patients’ fatigue.

The terminology of the FACIT-Fatigue was found to be clear, except for the item ‘I feel listless/washed out’ (item 3), which patients did not consistently understand as intended. Most patients linked ‘I feel listless/washed out’ to an absence of both physical and mental energy, but some interpreted it just as a lack of physical energy.

All patients found the response options provided by the FACIT-Fatigue to be suitable and sufficient. Conceptual relevance of the FACIT-Fatigue was supported by mapping of its items to the seven fatigue-related sub-components identified during concept elicitation [6] (Table 2).

**Table 2:** Mapping FACIT-Fatigue items to fatigue-related sub-components identified during concept elicitation

	Symptom concepts				Impact concepts		
FACIT-Fatigue item	Tiredness/need for sleep	Lack of energy	Weakness	Cognitive fatigue	Decreased ability to maintain social/familial/professional role	Decreased physical functioning	Frustration
1. I feel fatigued	✓	✓	✓	✓			
2. I feel weak all over			✓				
3. I feel listless/washed out		✓		✓			
4. I feel tired	✓						
5. I have trouble starting things because I am tired		✓		✓	✓	✓	
6. I have trouble finishing things because I am tired		✓		✓	✓	✓	
7. I have energy		✓					
8. I am able to do my usual activities					✓	✓	
9. I need to sleep during the day	✓						
10. I am too tired to eat						✓	
11. I need help doing my usual activities						✓	
12. I am frustrated by being too tired to do the things I want to do							✓
13. I have to limit my social activity because I am tired					✓		

*Abbreviation: FACIT-Fatigue* Functional Assessment of Chronic Illness Therapy-Fatigue scale

### Psychometric analysis in CLL

Baseline PRO data were available for 263 patients (85%) enrolled in the phase 3 ASCEND trial. Median age was 67 (range: 32–89) years and 67% were men. At baseline, 231 patients (88%) had an ECOG Performance Status score of 0 or 1, and 32 patients (12%) had an ECOG Performance Status score of 2. The baseline mean FACIT-Fatigue score was 35.27 (SD: 9.87) for the total scale, out of a possible maximum score of 52, and was 12.09 (SD: 4.34) and 23.18 (SD: 6.12) for the symptom and impact subscale, respectively. Mean FACIT-Fatigue scores ranged from 1.01 to 1.84 for symptom items and from 0.40 to 2.23 for impact items. Some ceiling effects (scoring ‘best possible health state’) were observed: the proportion of patients who answered ‘not at all’ (indicating that they did not have the symptom or impact) was above 25% for only one of the five symptom subscale items (item 3, ‘I feel listless/washed out’); however, more than 25% of patients answered ‘not at all’ for seven of the eight impact subscale items, indicating that there was low impact of fatigue on activities at baseline (Supplemental File 1). No floor effects (scoring ‘worst possible health state’) were observed (Supplemental File 1).

#### Confirmatory factor analysis

The one-factor solution (Table 3) showed acceptable fit based on the CFI (0.946), SRMR (0.066) and the loadings (all factor loadings > 0.30) and a poor fit based on the RMSEA (0.152; 95% confidence interval [CI]: 0.139–0.165). The individual subscales also showed good fit when examining the CFI and the loadings and a poor fit when examining the RMSEA. The bifactor model showed acceptable fit based on the CFI (0.973), SRMR (0.044) and a poor fit based on the RMSEA (0.120; 95% CI: 0.105–0.135).

**Table 3:** Confirmatory factor analysis

FACIT-Fatigue	RMSEA (90% CI)	SRMR	CFI	ω	ωH	ECV
Single factor modeling						
Symptom subscale	0.068 (0.005–0.123)	0.014	0.998	0.903		
Impact subscale	0.166 (0.143–0.190)	0.061	0.961	0.907		
Total scale	0.152 (0.139–0.165)	0.066	0.946	0.943		
Bifactor modeling	0.120 (0.105–0.135)	0.044	0.973	0.954	0.905	0.819

*Abbreviations: CI* confidence interval, *CFI* comparative fit index, *ECV* explained common variance, *FACIT-Fatigue* Functional Assessment of Chronic Illness Therapy-Fatigue scale, *RMSEA* root mean square error of approximation, *SRMR* standardized root mean square residual, *ω* omega, *ωH* omega hierarchical

Standard cutoff values: RMSEA < 0.06; SRMR < 0.08; CFI > 0.95 [17–20]

All factor loadings were statistically significant for the one factor (Supplemental File 2). For the bifactor model all factors were significant for the general factor, but for the sub-domain factors item ‘I have energy’ (item 7) loading on the fatigue symptom domain and item ‘I need to sleep during the day’ (item 9) on the fatigue impact domain were not significant (Supplemental File 3). Results from the bifactor model showed that almost all items had higher loadings on the general factor (range: 0.43 to 0.95) than on the two sub-domain factors (symptoms, range: −0.06 to 0.47; impacts, range: −0.21 to 0.66), supporting the essential unidimensionality of the FACIT-Fatigue. In addition, loadings on the general factor were very similar to the factor loadings of the single factor analysis.

#### Internal consistency reliability

Cronbach’s coefficient α was 0.87 (95% CI: 0.84–0.89) for the FACIT-Fatigue symptom subscale, 0.86 (95% CI: 0.83–0.88) for the impact subscale and 0.91 (95% CI: 0.90–0.93) for the total scale. McDonald’s ω was 0.94 for the FACIT-Fatigue total scale, 0.91 for the impact subscale and 0.90 for the symptom subscale. For the bifactor model ω coefficient was 0.95 and ωH was 0.91. A comparison of ωH (0.91) with ω (0.95) is critical. Here, we see that almost all of the reliable variance in total scores (0.91/0.95 = 0.96) can be attributed to the general factor, assumed to reflect individual differences on the trait of fatigue. Only 4% (ω - ωH) of the reliable variance in total scores can be attributed to the multidimensionality caused by the subgroup factors. ECV was 0.82, also indicating a quite strong general factor accounting for well over half the common variance. Most item-to-item correlations and all item-to-total correlations were moderate to strong (Spearman’s rank correlation coefficient *r* ≥ 0.30).

#### Construct validity

The FACIT-Fatigue symptom subscale, impact subscale and total scale scores correlated strongly with the EORTC QLQ-C30 global health status, physical function, role function and fatigue scale scores, and the EQ-VAS score (all Spearman’s *r* ≥ 0.50), demonstrating convergent validity (Table 4). Weak correlations (Spearman’s *r* < 0.30) were observed between the FACIT-Fatigue scales, and the EORTC QLQ-C30 insomnia, constipation and diarrhea scales, indicating that there was no relationship between fatigue and these symptoms, and supporting divergent validity (Table 3).

**Table 4:** Correlations to assess the convergent and divergent validity of the FACIT-Fatigue scales versus other measures

	Spearman’s *r*		
FACIT-Fatigue vs:	Symptom subscale	Impact subscale	Global Fatigue scale
EORTC QLQ-C30			
Global health status	0.66	0.64	0.69
Physical functioning	0.61	0.71	0.71
Role functioning	0.56	0.67	0.66
Emotional functioning	0.49	0.54	0.55
Cognitive functioning	0.37	0.52	0.48
Social functioning	0.47	0.57	0.55
Fatigue	−0.75	−0.75	−0.80
Nausea/vomiting	−0.33	−0.33	−0.35
Pain	−0.39	−0.43	−0.44
Dyspnea	−0.34	−0.32	−0.35
Insomnia	−0.28	−0.26	−0.28
Appetite loss	−0.39	−0.42	−0.44
Constipation	−0.13	−0.20	−0.18
Diarrhea	−0.18	−0.16	−0.17
Financial problems	−0.21	−0.31	−0.29
EQ-5D-5L			
Mobility	−0.40	−0.45	−0.46
Self-care	−0.19	−0.32	−0.29
Usual activities	−0.48	−0.61	−0.59
Pain/discomfort	−0.39	−0.38	−0.41
Anxiety/depression	−0.44	−0.47	−0.49
EQ-VAS score	0.61	0.60	0.64

Correlations are assessed based on absolute values. Strong correlations: absolute *r*_s_ ≥ 0.50; moderate correlation: absolute 0.30 ≤ *r*_s_ < 0.50; weak correlations: absolute 0.10 ≤ *r*_s_ < 0.30

*Abbreviations: EORTC QLQ-C30* European Organisation for Research and Treatment of Cancer Quality of Life Questionnaire-Core 30-questions, *EQ-5D-5L* 5-level, 5-dimension EuroQol questionnaire, *EQ-VAS* EuroQol global health visual analog scale, *FACIT-Fatigue* Functional Assessment of Chronic Illness Therapy-Fatigue scale, *r*_s_ Spearman’s *r*

#### Known-groups validity

**Fig. 1:**
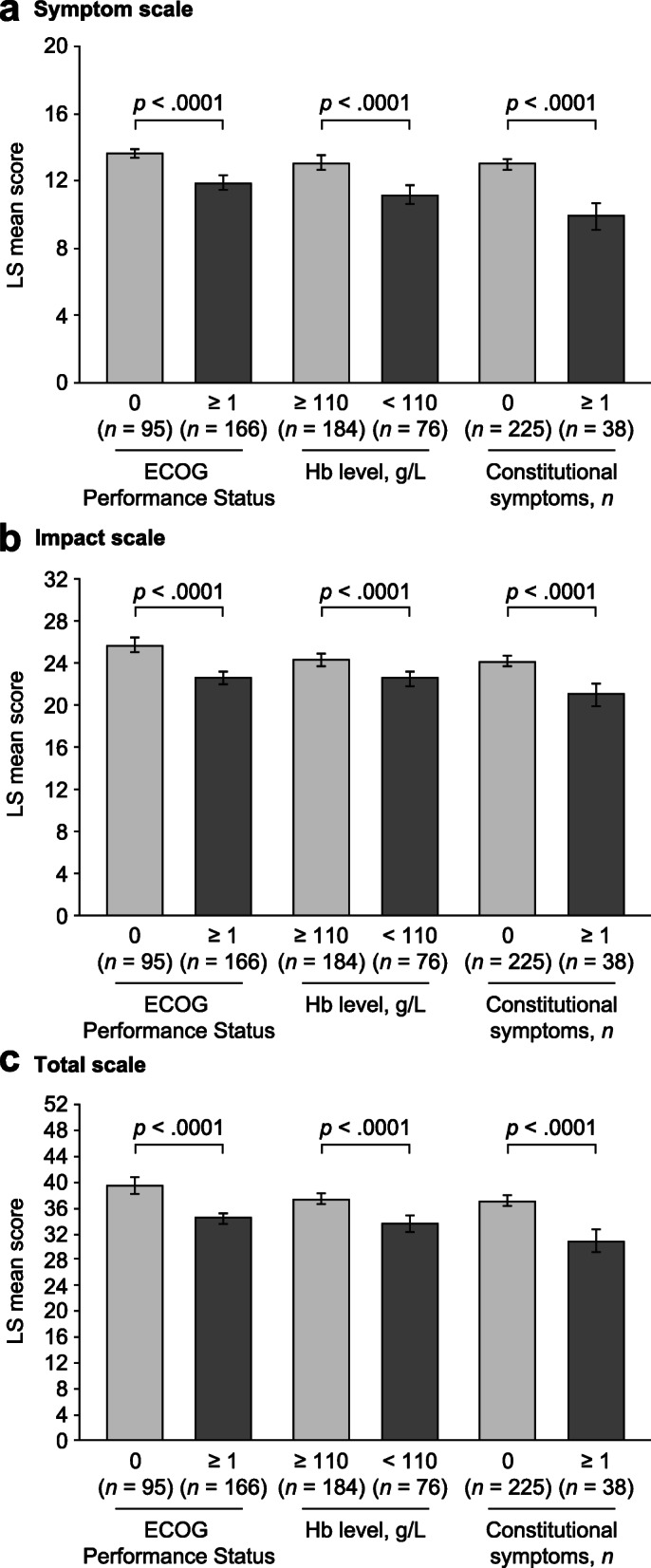
LS mean (SE) FACIT-Fatigue scores at baseline by known-groups. Information on ECOG Performance Status scores and Hb levels was unknown for two and three patients, respectively. Note that, because of the small sample size for ECOG Performance Status score 2, the known groups used here differ from the stratification of 0 or 1 vs 2 that were used as part of stratified randomization in the ASCEND trial. For two patients with ECOG Performance Status score < 2, information was missing on whether their ECOG Performance Status score was 0 or 1. *Abbreviations: ECOG* Eastern Cooperative Oncology Group, *FACIT-Fatigue* Functional Assessment of Chronic Illness Therapy-Fatigue scale, *Hb* hemoglobin, *LS* least-squares, *SE* standard error

Figure 1 shows the number of patients and mean FACIT-Fatigue scores at baseline by known groups. Known-groups validity of the FACIT-Fatigue scales was demonstrated by significant differences between groups defined by baseline ECOG Performance Status score, Hb level and constitutional symptoms (Fig. 1).

#### Defining the severity cut-off score

Substantial agreement was observed between clusters grouped by FACIT-Fatigue and EORTC QLQ-C30 scores and the severe fatigue population defined using a FACIT-Fatigue total score threshold of either 30 or 34 (Cohen’s kappa coefficients of 0.76 and 0.67, respectively).

## Discussion

This work demonstrates the validity and reliability of the FACIT-Fatigue, a PRO instrument that assesses fatigue-related symptoms and impacts, in patients with CLL and shows that it is fit for purpose in this population. Cognitive debriefing of the FACIT-Fatigue in patients with 1 L or R/R CLL, conducted as part of qualitative interviews, confirmed that the instrument items are easily understood, interpreted as intended, relevant to patients with CLL at different disease stages and comprehensive. An exception was item 10, ‘I am too tired to eat’, which was not considered to be highly pertinent. The item is targeted to very severe fatigue, which often people cannot relate to unless they have experienced it themselves. This is consistent with findings from FACIT-Fatigue validation studies in other disease settings such as iron-deficiency anemia, systemic lupus erythematosus and psoriatic arthritis [34–36]. However, patients thought that the item was still appropriate to retain.

The concept elicitation part of the qualitative interview study has been described previously [6]. It showed that fatigue is a key experience in patients with CLL that manifests as a variety of sub-components related to symptoms in CLL (tiredness/need for sleep; lack of energy; weakness; cognitive fatigue) and impacts (decreased ability to maintain social, familial or professional role; decreased physical functioning; frustration). In the current work, the FACIT-Fatigue items were shown to map well to the fatigue-related sub-components identified previously during concept elicitation, providing further support of the relevance and comprehensiveness of the FACIT-Fatigue in the CLL setting. Mean FACIT-Fatigue scores for patients with CLL were lower (worse) than general population scores [5, 13], indicating greater fatigue in the CLL population than the general population and providing further evidence that fatigue is a core component of CLL.

In the psychometric evaluation of phase 3 study data in patients with R/R CLL, CFA analysis and the examination of psychometrically informative bifactor-derived statistics supported the unidimensionality of the FACIT-Fatigue. More specifically, the calculation of ω and ωΗ indicated that a strong percentage of total score variance is attributable to the single general factor. As a consequence, we conclude that raw scores can essentially be assumed as indicators of the FACIT-Fatigue general factor and are not affected by the multidimensionality of the two subscales. The symptom and impact subscales could be distinguished as separate components that can be reported separately or combined as a total score, confirming previous analyses of the dimensionality of the FACIT-Fatigue [7]. In addition, our factor analysis findings, including strength of loadings and fit statistics, are consistent with the study of Cella et al. 2011 [7]. All three scales (symptom, impact, total) demonstrated good internal consistency, reliability, construct validity and known-groups validity, providing choice depending on purpose of use. The three FACIT-Fatigue scales differentiated between groups defined by disease severity indicators. Patients who were fully active without restrictions (ECOG Performance Status score 0) scored significantly better (i.e. less fatigue) on all scales than patients who were ambulatory but restricted in physically strenuous activity or unable to carry out any work activities (ECOG Performance Status score 1 or 2). Similarly, patients with no anemia or mild anemia (Hb ≥ 110 g/L) scored significantly better on all three scales than those with moderate or severe anemia (Hb < 110 g/L), and patients with no constitutional CLL symptoms scored significantly better than those with at least one such symptom.

In addition to demonstrating known-groups validity, the ability of the three FACIT-Fatigue scales to differentiate between groups based on disease severity indicators supports their relevance for use in clinical trials. Cluster analysis supported the previously identified FACIT-Fatigue total score thresholds of 30 and 34 to define a severe fatigue population.

A limitation of our work is that test–retest reliability of the FACIT-Fatigue was not assessed, because there was only one assessment time point (at baseline) before study drug initiation and no acceptable independent measures to identify patients remaining in a stable condition were used during the study. Interviewed patients were younger than those in the ASCEND trial (median age 58 years vs 67 years). Psychometric properties were evaluated in patients with R/R CLL only. Patients enrolled in a pivotal phase 3 CLL treatment trial may have less comorbidity than patients treated for CLL in the real world. Additional studies are needed to determine the instrument’s responsiveness to change [20].

This work had important strengths, including the rigorous methodologies used, the comprehensive assessments conducted, an appropriate sample size for cognitive debriefing and the large sample size available for the psychometric evaluation.

## Conclusions

Content validity assessment in patients with 1 L or R/R CLL and psychometric evaluation in patients with R/R CLL demonstrated that the FACIT-Fatigue has good psychometric properties and is fit for purpose in CLL. The three scoring options for the FACIT-Fatigue (symptom, impact, total) have similar reliability and validity, enabling choice depending on purpose of use. Results support the use of the FACIT-Fatigue in patients with 1 L or R/R CLL in the clinical trial setting.

## Supplementary Information

**Additional file 1 **: **Supplemental File 1**. Proportion of patients with response ‘not at all’ and ‘very much’ for FACIT-Fatigue items.

**Additional file 2 : Supplemental File 2**. Factor loadings for the one factor models of the FACIT-Fatigue scale, Impact subscale and Symptom subscale.

**Additional file 3 **: **Supplemental File 3**. Factor loadings for the bifactor model of the FACIT-Fatigue.

## Data Availability

Acerta Pharma, a member of the AstraZeneca Group, is committed to data transparency and will consider data sharing requests on a case-by-case basis. Any requests for de-identified patient data can be submitted to Acerta Pharma 3 months post-publication and ending 5 years following article publication with the intent-to-achieve aims of the original proposal. In addition, Acerta Pharma will provide the study protocol, statistical analysis plan, and informed consent form, as well as post results on clinicaltrials.gov, as required.

## Abbreviations

1 L: First-line
CFA: Confirmatory factor analysis
CFI: Comparative fit index
CI: Confidence interval
CLL: Chronic lymphocytic leukemia
ECOG: Eastern Cooperative Oncology Group
EORTC QLQ-30: European Organisation for Research and Treatment of Cancer Quality of Life Questionnaire-Core 30-questions
EQ-5D-5L: 5-level 5-dimension Euro Qol questionnaire
EQ-VAS: EuroQol visual analogue scale
FACIT-Fatigue: Functional Assessment of Chronic Illness Therapy-Fatigue scale
Hb: Hemoglobin
LS: Least-squares
PRO: Patient-reported outcome
R/R: Relapsed or refractory
RMSEA: Root mean square error of approximation
SD: Standard deviation
SE: Standard error
SRMR: Standardized root mean square residual
