# Supplementary material for: Content validity and psychometric evaluation of the Functional Assessment of Chronic Illness Therapy-Fatigue scale in patients with chronic lymphocytic leukemia
Source: J Patient Rep Outcomes. 2021 Mar 11;5:27. doi: 10.1186/s41687-021-00294-1 (PMC7952480; doi:10.1186/s41687-021-00294-1)
Supplement: Supplementary file 1 — Additional file 1 : Supplemental File 1. Proportion of patients with response ‘not at all’ and ‘very much’ for FACIT-Fatigue items. [file 41687_2021_294_MOESM1_ESM.docx]

Content validity and psychometric evaluation of the Functional Assessment of Chronic Illness Therapy-Fatigue scale in patients with chronic lymphocytic leukemia

## Supplemental File 1

Proportion of patients with response ‘not at all’ and ‘very much’ for FACIT-Fatigue items

| FACIT-Fatigue item | Not at all (ceiling effect), % | Very much (floor effect), % |
| --- | --- | --- |
| 1. I feel fatigued | 19.77 | 3.42 |
| 2. I feel weak all over | 0.00 | 0.00 |
| 3. I feel listless/washed out | 42.21 | 2.66 |
| 4. I feel tired | 15.59 | 5.32 |
| 5. I have trouble starting things because I am tired | 33.08 | 2.28 |
| 6. I have trouble finishing things because I am tired | 32.70 | 2.28 |
| 7. I have energy | 9.51 | 3.42 |
| 8. I am able to do my usual activities | 11.03 | 16.35 |
| 9. I need to sleep during the day | 25.86 | 2.28 |
| 10. I am too tired to eat | 74.90 | 0.00 |
| 11. I need help doing my usual activities | 67.68 | 1.52 |
| 12. I am frustrated by being too tired to do the things I want to do | 40.68 | 5.70 |
| 13. I have to limit my social activity because I am tired | 41.06 | 6.08 |

*Abbreviations: FACIT-Fatigue* Functional Assessment of Chronic Illness Therapy-Fatigue scale
