# Supplementary material for: Content validity and psychometric evaluation of the Functional Assessment of Chronic Illness Therapy-Fatigue scale in patients with chronic lymphocytic leukemia
Source: J Patient Rep Outcomes. 2021 Mar 11;5:27. doi: 10.1186/s41687-021-00294-1 (PMC7952480; doi:10.1186/s41687-021-00294-1)
Supplement: Supplementary file 2 — Additional file 2 : Supplemental File 2. Factor loadings for the one factor models of the FACIT-Fatigue scale, Impact subscale and Symptom subscale. [file 41687_2021_294_MOESM2_ESM.docx]

Content validity and psychometric evaluation of the Functional Assessment of Chronic Illness Therapy-Fatigue scale in patients with chronic lymphocytic leukemia

## Supplemental File 2

Factor loadings for the one factor models of the FACIT-Fatigue scale, Impact subscale and Symptom subscale.

| FACIT-Fatigue item | Total scale | Impact subscale | Symptom subscale |
| --- | --- | --- | --- |
| 1. I feel fatigued | 0.833 |  | 0.871 |
| 2. I feel weak all over | 0.883 |  | 0.924 |
| 3. I feel listless/washed out | 0.740 |  | 0.756 |
| 4. I feel tired | 0.889 |  | 0.911 |
| 5. I have trouble starting things because I am tired | 0.922 | 0.933 |  |
| 6. I have trouble finishing things because I am tired | 0.883 | 0.899 |  |
| 7. I have energy | 0.595 |  | 0.524 |
| 8. I am able to do my usual activities | 0.477 | 0.473 |  |
| 9. I need to sleep during the day | 0.425 | 0.429 |  |
| 10. I am too tired to eat | 0.789 | 0.784 |  |
| 11. I need help doing my usual activities | 0.577 | 0.654 |  |
| 12. I am frustrated by being too tired to do the things I want to do | 0.789 | 0.822 |  |
| 13. I have to limit my social activity because I am tired | 0.818 | 0.839 |  |

*Abbreviations: FACIT-Fatigue* Functional Assessment of Chronic Illness Therapy-Fatigue scale
