# Supplementary material for: Content validity and psychometric evaluation of the Functional Assessment of Chronic Illness Therapy-Fatigue scale in patients with chronic lymphocytic leukemia
Source: J Patient Rep Outcomes. 2021 Mar 11;5:27. doi: 10.1186/s41687-021-00294-1 (PMC7952480; doi:10.1186/s41687-021-00294-1)
Supplement: Supplementary file 3 — Additional file 3 : Supplemental File 3. Factor loadings for the bifactor model of the FACIT-Fatigue. [file 41687_2021_294_MOESM3_ESM.docx]

Content validity and psychometric evaluation of the Functional Assessment of Chronic Illness Therapy-Fatigue scale in patients with chronic lymphocytic leukemia

## Supplemental File 3

Factor loadings for the bifactor model of the FACIT-Fatigue.

| FACIT-Fatigue item | General Factor | Symptom subdomain | Impact subdomain |
| --- | --- | --- | --- |
| 1. I feel fatigued | 0.742 | 0.474 |  |
| 2. I feel weak all over | 0.794 | 0.470 |  |
| 3. I feel listless/washed out | 0.702 | 0.282 |  |
| 4. I feel tired | 0.823 | 0.394 |  |
| 5. I have trouble starting things because I am tired | 0.946 |  | -0.160 |
| 6. I have trouble finishing things because I am tired | 0.892 |  | -0.201 |
| 7. I have energy | 0.630 | -0.064 |  |
| 8. I am able to do my usual activities | 0.462 |  | 0.415 |
| 9. I need to sleep during the day | 0.432 |  | 0.054 |
| 10. I am too tired to eat | 0.782 |  | 0.249 |
| 11. I need help doing my usual activities | 0.523 |  | 0.663 |
| 12. I am frustrated by being too tired to do the things I want to do | 0.795 |  | 0.218 |
| 13. I have to limit my social activity because I am tired | 0.824 |  | 0.279 |

*Abbreviations: FACIT-Fatigue* Functional Assessment of Chronic Illness Therapy-Fatigue scale
